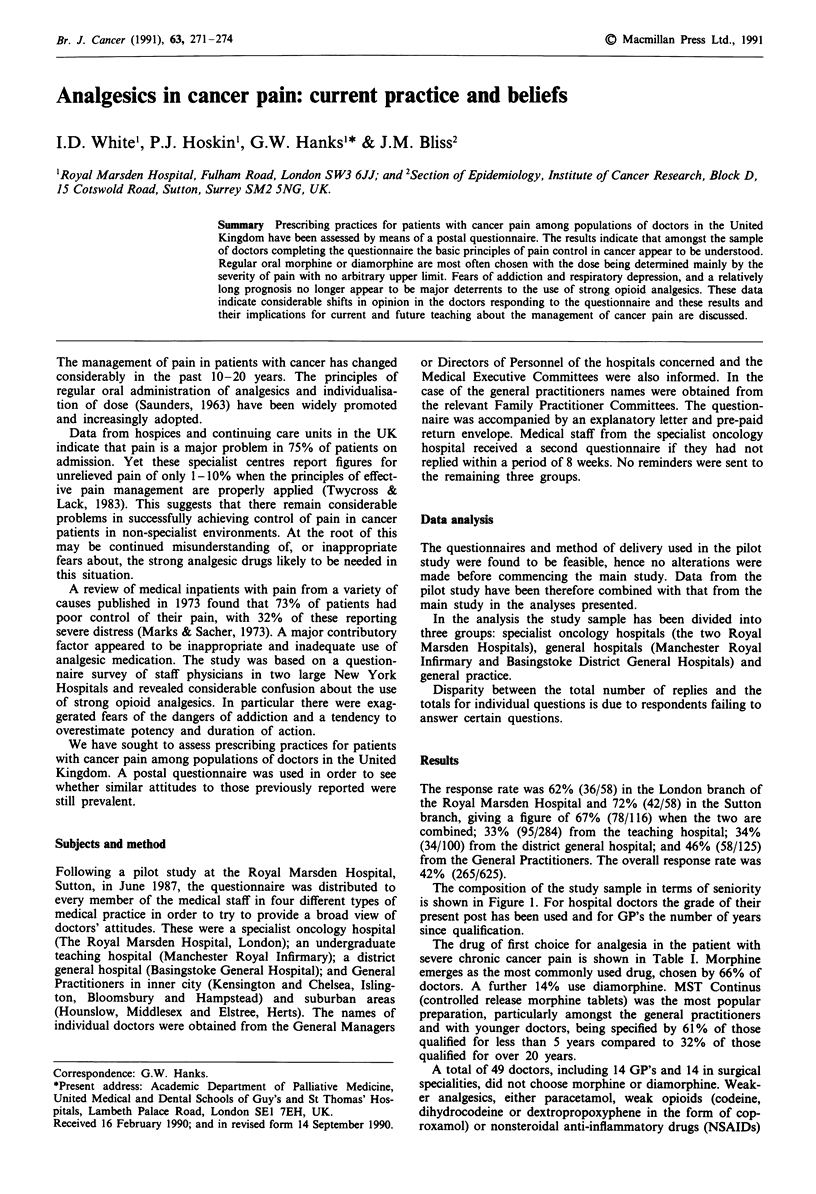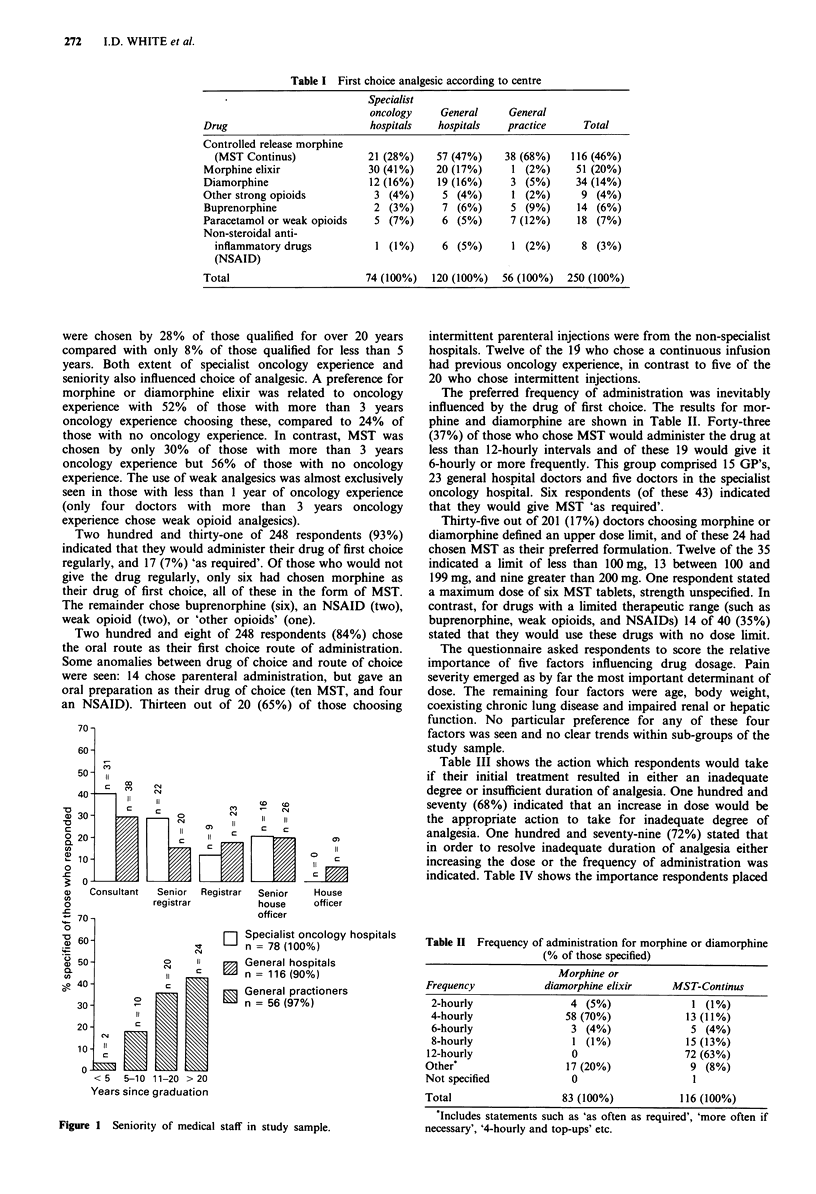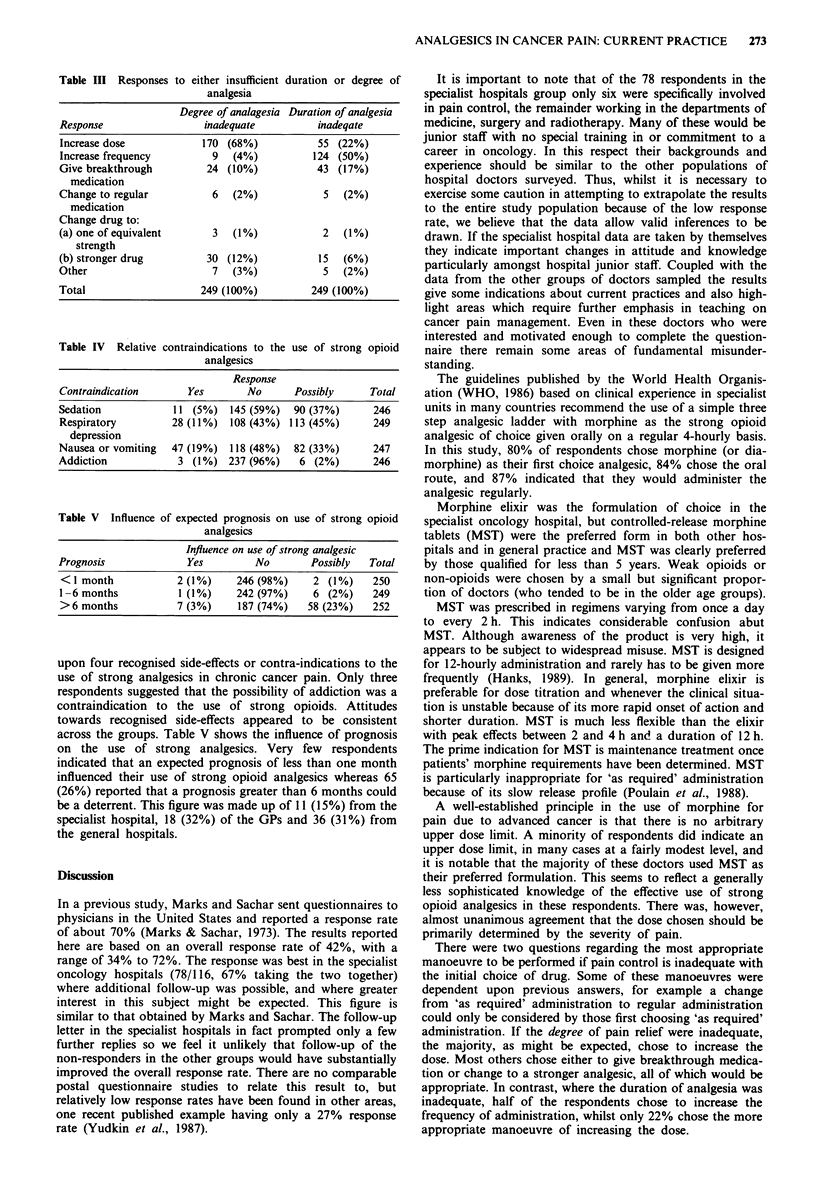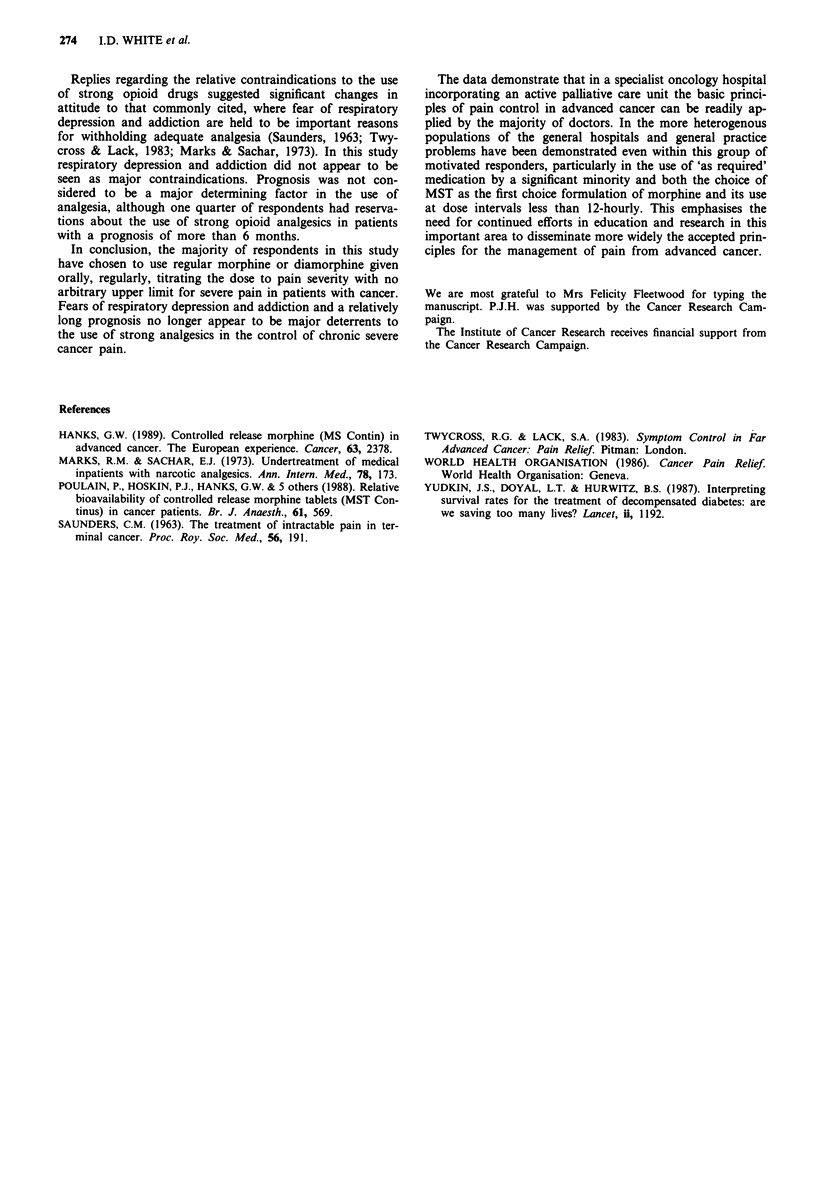# Analgesics in cancer pain: current practice and beliefs.

**DOI:** 10.1038/bjc.1991.63

**Published:** 1991-02

**Authors:** I. D. White, P. J. Hoskin, G. W. Hanks, J. M. Bliss

**Affiliations:** Royal Marsden Hospital, London, UK.

## Abstract

Prescribing practices for patients with cancer pain among populations of doctors in the United Kingdom have been assessed by means of a postal questionnaire. The results indicate that amongst the sample of doctors completing the questionnaire the basic principles of pain control in cancer appear to be understood. Regular oral morphine or diamorphine are most often chosen with the dose being determined mainly by the severity of pain with no arbitrary upper limit. Fears of addiction and respiratory depression, and a relatively long prognosis no longer appear to be major deterrents to the use of strong opioid analgesics. These data indicate considerable shifts in opinion in the doctors responding to the questionnaire and these results and their implications for current and future teaching about the management of cancer pain are discussed.


Br. J. Cancer (1991), 63, 271–274 © Macmillan Press Ltd., 1991

### Analgesics in cancer pain: current practice and beliefs

I.D. White[1], P.J. Hoskin[1], G.W. Hanks[1]* & J.M. Bliss[2]

[1]*Royal Marsden Hospital, Fulham Road, London SW3 6JJ; and* [2]*Section of Epidemiology, Institute of Cancer Research, Block D, 15 Cotswold Road, Sutton, Surrey SM2 5NG, UK.*

**Summary** Prescribing practices for patients with cancer pain among populations of doctors in the United Kingdom have been assessed by means of a postal questionnaire. The results indicate that amongst the sample of doctors completing the questionnaire the basic principles of pain control in cancer appear to be understood. Regular oral morphine or diamorphine are most often chosen with the dose being determined mainly by the severity of pain with no arbitrary upper limit. Fears of addiction and respiratory depression, and a relatively long prognosis no longer appear to be major deterrents to the use of strong opioid analgesics. These data indicate considerable shifts in opinion in the doctors responding to the questionnaire and these results and their implications for current and future teaching about the management of cancer pain are discussed.

The management of pain in patients with cancer has changed considerably in the past 10–20 years. The principles of regular oral administration of analgesics and individualisation of dose (Saunders, 1963) have been widely promoted and increasingly adopted.

Data from hospices and continuing care units in the UK indicate that pain is a major problem in 75% of patients on admission. Yet these specialist centres report figures for unrelieved pain of only 1–10% when the principles of effective pain management are properly applied (Twycross & Lack, 1983). This suggests that there remain considerable problems in successfully achieving control of pain in cancer patients in non-specialist environments. At the root of this may be continued misunderstanding of, or inappropriate fears about, the strong analgesic drugs likely to be needed in this situation.

A review of medical inpatients with pain from a variety of causes published in 1973 found that 73% of patients had poor control of their pain, with 32% of these reporting severe distress (Marks & Sacher, 1973). A major contributory factor appeared to be inappropriate and inadequate use of analgesic medication. The study was based on a questionnaire survey of staff physicians in two large New York Hospitals and revealed considerable confusion about the use of strong opioid analgesics. In particular there were exaggerated fears of the dangers of addiction and a tendency to overestimate potency and duration of action.

We have sought to assess prescribing practices for patients with cancer pain among populations of doctors in the United Kingdom. A postal questionnaire was used in order to see whether similar attitudes to those previously reported were still prevalent.

### Subjects and method

Following a pilot study at the Royal Marsden Hospital, Sutton, in June 1987, the questionnaire was distributed to every member of the medical staff in four different types of medical practice in order to try to provide a broad view of doctors' attitudes. These were a specialist oncology hospital (The Royal Marsden Hospital, London); an undergraduate teaching hospital (Manchester Royal Infirmary); a district general hospital (Basingstoke General Hospital); and General Practitioners in inner city (Kensington and Chelsea, Islington, Bloomsbury and Hampstead) and suburban areas (Hounslow, Middlesex and Elstree, Herts). The names of individual doctors were obtained from the General Managers or Directors of Personnel of the hospitals concerned and the Medical Executive Committees were also informed. In the case of the general practitioners names were obtained from the relevant Family Practitioner Committees. The questionnaire was accompanied by an explanatory letter and pre-paid return envelope. Medical staff from the specialist oncology hospital received a second questionnaire if they had not replied within a period of 8 weeks. No reminders were sent to the remaining three groups.

### Data analysis

The questionnaires and method of delivery used in the pilot study were found to be feasible, hence no alterations were made before commencing the main study. Data from the pilot study have been therefore combined with that from the main study in the analyses presented.

In the analysis the study sample has been divided into three groups: specialist oncology hospitals (the two Royal Marsden Hospitals), general hospitals (Manchester Royal Infirmary and Basingstoke District General Hospitals) and general practice.

Disparity between the total number of replies and the totals for individual questions is due to respondents failing to answer certain questions.

### Results

The response rate was 62% (36/58) in the London branch of the Royal Marsden Hospital and 72% (42/58) in the Sutton branch, giving a figure of 67% (78/116) when the two are combined; 33% (95/284) from the teaching hospital; 34% (34/100) from the district general hospital; and 46% (58/125) from the General Practitioners. The overall response rate was 42% (265/625).

The composition of the study sample in terms of seniority is shown in Figure 1. For hospital doctors the grade of their present post has been used and for GP's the number of years since qualification.

The drug of first choice for analgesia in the patient with severe chronic cancer pain is shown in Table I. Morphine emerges as the most commonly used drug, chosen by 66% of doctors. A further 14% use diamorphine. MST Continus (controlled release morphine tablets) was the most popular preparation, particularly amongst the general practitioners and with younger doctors, being specified by 61% of those qualified for less than 5 years compared to 32% of those qualified for over 20 years.

A total of 49 doctors, including 14 GP's and 14 in surgical specialities, did not choose morphine or diamorphine. Weaker analgesics, either paracetamol, weak opioids (codeine, dihydrocodeine or dextropropoxyphene in the form of coproxamol) or nonsteroidal anti-inflammatory drugs (NSAIDs)

Correspondence: G.W. Hanks.
*Present address: Academic Department of Palliative Medicine, United Medical and Dental Schools of Guy's and St Thomas' Hospitals, Lambeth Palace Road, London SE1 7EH, UK.
Received 16 February 1990; and in revised form 14 September 1990.

**Table I** First choice analgesic according to centre

| Drug | Specialist oncology hospitals | General hospitals | General practice | Total |
|---|---|---|---|---|
| Controlled release morphine (MST Continus) | 21 (28%) | 57 (47%) | 38 (68%) | 116 (46%) |
| Morphine elixir | 30 (41%) | 20 (17%) | 1 (2%) | 51 (20%) |
| Diamorphine | 12 (16%) | 19 (16%) | 3 (5%) | 34 (14%) |
| Other strong opioids | 3 (4%) | 5 (4%) | 1 (2%) | 9 (4%) |
| Buprenorphine | 2 (3%) | 7 (6%) | 5 (9%) | 14 (6%) |
| Paracetamol or weak opioids | 5 (7%) | 6 (5%) | 7 (12%) | 18 (7%) |
| Non-steroidal anti-inflammatory drugs (NSAID) | 1 (1%) | 6 (5%) | 1 (2%) | 8 (3%) |
| Total | 74 (100%) | 120 (100%) | 56 (100%) | 250 (100%) |

were chosen by 28% of those qualified for over 20 years compared with only 8% of those qualified for less than 5 years. Both extent of specialist oncology experience and seniority also influenced choice of analgesic. A preference for morphine or diamorphine elixir was related to oncology experience with 52% of those with more than 3 years oncology experience choosing these, compared to 24% of those with no oncology experience. In contrast, MST was chosen by only 30% of those with more than 3 years oncology experience but 56% of those with no oncology experience. The use of weak analgesics was almost exclusively seen in those with less than 1 year of oncology experience (only four doctors with more than 3 years oncology experience chose weak opioid analgesics).

Two hundred and thirty-one of 248 respondents (93%) indicated that they would administer their drug of first choice regularly, and 17 (7%) 'as required'. Of those who would not give the drug regularly, only six had chosen morphine as their drug of first choice, all of these in the form of MST. The remainder chose buprenorphine (six), an NSAID (two), weak opioid (two), or 'other opioids' (one).

Two hundred and eight of 248 respondents (84%) chose the oral route as their first choice route of administration. Some anomalies between drug of choice and route of choice were seen: 14 chose parenteral administration, but gave an oral preparation as their drug of choice (ten MST, and four an NSAID). Thirteen out of 20 (65%) of those choosing intermittent parenteral injections were from the non-specialist hospitals. Twelve of the 19 who chose a continuous infusion had previous oncology experience, in contrast to five of the 20 who chose intermittent injections.

**Figure 1** Seniority of medical staff in study sample.

The preferred frequency of administration was inevitably influenced by the drug of first choice. The results for morphine and diamorphine are shown in Table II. Forty-three (37%) of those who chose MST would administer the drug at less than 12-hourly intervals and of these 19 would give it 6-hourly or more frequently. This group comprised 15 GP's, 23 general hospital doctors and five doctors in the specialist oncology hospital. Six respondents (of these 43) indicated that they would give MST 'as required'.

Thirty-five out of 201 (17%) doctors choosing morphine or diamorphine defined an upper dose limit, and of these 24 had chosen MST as their preferred formulation. Twelve of the 35 indicated a limit of less than 100 mg, 13 between 100 and 199 mg, and nine greater than 200 mg. One respondent stated a maximum dose of six MST tablets, strength unspecified. In contrast, for drugs with a limited therapeutic range (such as buprenorphine, weak opioids, and NSAIDs) 14 of 40 (35%) stated that they would use these drugs with no dose limit.

The questionnaire asked respondents to score the relative importance of five factors influencing drug dosage. Pain severity emerged as by far the most important determinant of dose. The remaining four factors were age, body weight, coexisting chronic lung disease and impaired renal or hepatic function. No particular preference for any of these four factors was seen and no clear trends within sub-groups of the study sample.

Table III shows the action which respondents would take if their initial treatment resulted in either an inadequate degree or insufficient duration of analgesia. One hundred and seventy (68%) indicated that an increase in dose would be the appropriate action to take for inadequate degree of analgesia. One hundred and seventy-nine (72%) stated that in order to resolve inadequate duration of analgesia either increasing the dose or the frequency of administration was indicated. Table IV shows the importance respondents placed

**Table II** Frequency of administration for morphine or diamorphine (% of those specified)

| Frequency | Morphine or diamorphine elixir | MST-Continus |
|---|---|---|
| 2-hourly | 4 (5%) | 1 (1%) |
| 4-hourly | 58 (70%) | 13 (11%) |
| 6-hourly | 3 (4%) | 5 (4%) |
| 8-hourly | 1 (1%) | 15 (13%) |
| 12-hourly | 0 | 72 (63%) |
| Other* | 17 (20%) | 9 (8%) |
| Not specified | 0 | 1 |
| Total | 83 (100%) | 116 (100%) |

*Includes statements such as 'as often as required', 'more often if necessary', '4-hourly and top-ups' etc.

**Table III** Responses to either insufficient duration or degree of analgesia

| Response | Degree of analagesia inadequate | Duration of analgesia inadeqate |
|---|---|---|
| Increase dose | 170 (68%) | 55 (22%) |
| Increase frequency | 9 (4%) | 124 (50%) |
| Give breakthrough medication | 24 (10%) | 43 (17%) |
| Change to regular medication | 6 (2%) | 5 (2%) |
| Change drug to: | | |
| (a) one of equivalent strength | 3 (1%) | 2 (1%) |
| (b) stronger drug | 30 (12%) | 15 (6%) |
| Other | 7 (3%) | 5 (2%) |
| Total | 249 (100%) | 249 (100%) |

**Table IV** Relative contraindications to the use of strong opioid analgesics

| Contraindication | Yes | Response No | Possibly | Total |
|---|---|---|---|---|
| Sedation | 11 (5%) | 145 (59%) | 90 (37%) | 246 |
| Respiratory depression | 28 (11%) | 108 (43%) | 113 (45%) | 249 |
| Nausea or vomiting | 47 (19%) | 118 (48%) | 82 (33%) | 247 |
| Addiction | 3 (1%) | 237 (96%) | 6 (2%) | 246 |

**Table V** Influence of expected prognosis on use of strong opioid analgesics

| Prognosis | Influence on use of strong analgesic Yes | No | Possibly | Total |
|---|---|---|---|---|
| <1 month | 2 (1%) | 246 (98%) | 2 (1%) | 250 |
| 1–6 months | 1 (1%) | 242 (97%) | 6 (2%) | 249 |
| >6 months | 7 (3%) | 187 (74%) | 58 (23%) | 252 |

upon four recognised side-effects or contra-indications to the use of strong analgesics in chronic cancer pain. Only three respondents suggested that the possibility of addiction was a contraindication to the use of strong opioids. Attitudes towards recognised side-effects appeared to be consistent across the groups. Table V shows the influence of prognosis on the use of strong analgesics. Very few respondents indicated that an expected prognosis of less than one month influenced their use of strong opioid analgesics whereas 65 (26%) reported that a prognosis greater than 6 months could be a deterrent. This figure was made up of 11 (15%) from the specialist hospital, 18 (32%) of the GPs and 36 (31%) from the general hospitals.

### Discussion

In a previous study, Marks and Sachar sent questionnaires to physicians in the United States and reported a response rate of about 70% (Marks & Sachar, 1973). The results reported here are based on an overall response rate of 42%, with a range of 34% to 72%. The response was best in the specialist oncology hospitals (78/116, 67% taking the two together) where additional follow-up was possible, and where greater interest in this subject might be expected. This figure is similar to that obtained by Marks and Sachar. The follow-up letter in the specialist hospitals in fact prompted only a few further replies so we feel it unlikely that follow-up of the non-responders in the other groups would have substantially improved the overall response rate. There are no comparable postal questionnaire studies to relate this result to, but relatively low response rates have been found in other areas, one recent published example having only a 27% response rate (Yudkin *et al.*, 1987).

It is important to note that of the 78 respondents in the specialist hospitals group only six were specifically involved in pain control, the remainder working in the departments of medicine, surgery and radiotherapy. Many of these would be junior staff with no special training in or commitment to a career in oncology. In this respect their backgrounds and experience should be similar to the other populations of hospital doctors surveyed. Thus, whilst it is necessary to exercise some caution in attempting to extrapolate the results to the entire study population because of the low response rate, we believe that the data allow valid inferences to be drawn. If the specialist hospital data are taken by themselves they indicate important changes in attitude and knowledge particularly amongst hospital junior staff. Coupled with the data from the other groups of doctors sampled the results give some indications about current practices and also highlight areas which require further emphasis in teaching on cancer pain management. Even in these doctors who were interested and motivated enough to complete the questionnaire there remain some areas of fundamental misunderstanding.

The guidelines published by the World Health Organisation (WHO, 1986) based on clinical experience in specialist units in many countries recommend the use of a simple three step analgesic ladder with morphine as the strong opioid analgesic of choice given orally on a regular 4-hourly basis. In this study, 80% of respondents chose morphine (or diamorphine) as their first choice analgesic, 84% chose the oral route, and 87% indicated that they would administer the analgesic regularly.

Morphine elixir was the formulation of choice in the specialist oncology hospital, but controlled-release morphine tablets (MST) were the preferred form in both other hospitals and in general practice and MST was clearly preferred by those qualified for less than 5 years. Weak opioids or non-opioids were chosen by a small but significant proportion of doctors (who tended to be in the older age groups).

MST was prescribed in regimens varying from once a day to every 2 h. This indicates considerable confusion abut MST. Although awareness of the product is very high, it appears to be subject to widespread misuse. MST is designed for 12-hourly administration and rarely has to be given more frequently (Hanks, 1989). In general, morphine elixir is preferable for dose titration and whenever the clinical situation is unstable because of its more rapid onset of action and shorter duration. MST is much less flexible than the elixir with peak effects between 2 and 4 h and a duration of 12 h. The prime indication for MST is maintenance treatment once patients' morphine requirements have been determined. MST is particularly inappropriate for 'as required' administration because of its slow release profile (Poulain *et al.*, 1988).

A well-established principle in the use of morphine for pain due to advanced cancer is that there is no arbitrary upper dose limit. A minority of respondents did indicate an upper dose limit, in many cases at a fairly modest level, and it is notable that the majority of these doctors used MST as their preferred formulation. This seems to reflect a generally less sophisticated knowledge of the effective use of strong opioid analgesics in these respondents. There was, however, almost unanimous agreement that the dose chosen should be primarily determined by the severity of pain.

There were two questions regarding the most appropriate manoeuvre to be performed if pain control is inadequate with the initial choice of drug. Some of these manoeuvres were dependent upon previous answers, for example a change from 'as required' administration to regular administration could only be considered by those first choosing 'as required' administration. If the *degree* of pain relief were inadequate, the majority, as might be expected, chose to increase the dose. Most others chose either to give breakthrough medication or change to a stronger analgesic, all of which would be appropriate. In contrast, where the duration of analgesia was inadequate, half of the respondents chose to increase the frequency of administration, whilst only 22% chose the more appropriate manoeuvre of increasing the dose.

Replies regarding the relative contraindications to the use of strong opioid drugs suggested significant changes in attitude to that commonly cited, where fear of respiratory depression and addiction are held to be important reasons for withholding adequate analgesia (Saunders, 1963; Twycross & Lack, 1983; Marks & Sachar, 1973). In this study respiratory depression and addiction did not appear to be seen as major contraindications. Prognosis was not considered to be a major determining factor in the use of analgesia, although one quarter of respondents had reservations about the use of strong opioid analgesics in patients with a prognosis of more than 6 months.

In conclusion, the majority of respondents in this study have chosen to use regular morphine or diamorphine given orally, regularly, titrating the dose to pain severity with no arbitrary upper limit for severe pain in patients with cancer. Fears of respiratory depression and addiction and a relatively long prognosis no longer appear to be major deterrents to the use of strong analgesics in the control of chronic severe cancer pain.

The data demonstrate that in a specialist oncology hospital incorporating an active palliative care unit the basic principles of pain control in advanced cancer can be readily applied by the majority of doctors. In the more heterogenous populations of the general hospitals and general practice problems have been demonstrated even within this group of motivated responders, particularly in the use of 'as required' medication by a significant minority and both the choice of MST as the first choice formulation of morphine and its use at dose intervals less than 12-hourly. This emphasises the need for continued efforts in education and research in this important area to disseminate more widely the accepted principles for the management of pain from advanced cancer.

We are most grateful to Mrs Felicity Fleetwood for typing the manuscript. P.J.H. was supported by the Cancer Research Campaign.

The Institute of Cancer Research receives financial support from the Cancer Research Campaign.

### References

HANKS, G.W. (1989). Controlled release morphine (MS Contin) in advanced cancer. The European experience. *Cancer*, **63**, 2378.

MARKS, R.M. & SACHAR, E.J. (1973). Undertreatment of medical inpatients with narcotic analgesics. *Ann. Intern. Med.*, **78**, 173.

POULAIN, P., HOSKIN, P.J., HANKS, G.W. & 5 others (1988). Relative bioavailability of controlled release morphine tablets (MST Continus) in cancer patients. *Br. J. Anaesth.*, **61**, 569.

SAUNDERS, C.M. (1963). The treatment of intractable pain in terminal cancer. *Proc. Roy. Soc. Med.*, **56**, 191.

TWYCROSS, R.G. & LACK, S.A. (1983). *Symptom Control in Far Advanced Cancer: Pain Relief.* Pitman: London.

WORLD HEALTH ORGANISATION (1986). *Cancer Pain Relief.* World Health Organisation: Geneva.

YUDKIN, J.S., DOYAL, L.T. & HURWITZ, B.S. (1987). Interpreting survival rates for the treatment of decompensated diabetes: are we saving too many lives? *Lancet*, **ii**, 1192.